# Partial Retention of the Placenta after Labor

**Published:** 1879-04

**Authors:** B. B. Browne

**Affiliations:** Baltimore, Md.


### Selections.

---

### PARTIAL RETENTION OF THE PLACENTA AFTER LABOR.

By B. B. BROWNE, M.D., Baltimore, Md.

Partial retention of the placenta may be caused by morbid adhesions between the placental and uterine tissues. But more frequently it occurs from a portion of the placenta being inadvertently left behind by the attendant in the delivery of the afterbirth.

A very interesting and instructive specimen of this latter kind was exhibited to the society by Dr. Coskery at the meeting of December 6th. Dr. Coskery made the *post-mortem* in the case, which occurred in the practice of another physician, and found in the uterus a portion of decomposing placenta about six inches long and three inches wide. Death was supposed to have been caused by peritonitis, and there was no suspicion that there was any retention of the placenta, as the uterus had apparently contracted satisfactorily after labor.

The following case of complete retention of the placenta occurred in my practice in 1869:

Mrs. M., about thirty-six years of age, the mother of several children, was enormously distended before labor; her limbs and face were also much swollen. On June 27 the membranes ruptured, and a very large amount of amniotic fluid was passed—more, I think, than I have even seen come from any six women; the bed was completely saturated, and the floor of the room was covered with water.

She had very weak pains, but in a short time gave birth to still-born twins. There was no hemorrhage, although there was complete inertia of the womb. In attempting to remove the placenta, I found that it was impossible to withdraw it. I then introduced my hand into the uterus, and with the other hand over the abdomen endeavored to separate the placenta from the uterus, but only succeeded in getting away some few fragments. Although large doses of fluid extract of ergot were given, there was not a particle of contraction in the uterus. Having completely failed to extract it, and thinking that some one else might succeed better than myself, I called on Dr. H. P. C. Wilson, who saw the case with me about ten hours after the birth of the children. For nearly an hour he made every effort to separate it, and finally concluded it could not be done without rupturing the uterus. He then advised that no further effort should be made to remove it, but that we should trust altogether to injections of hot water and carbolic acid into the vagina and uterus. These injections I administered myself two and three times daily. Full doses of quinine and opium were given internally. On the fourth day the placenta sloughed off from the uterus, and the patient was glad to get rid of it as quickly as possible. There is perhaps no stench more offensive than that of a placenta that has decomposed in utero. From that time she rapidly improved, and had no further trouble.

It is reasonable to believe that in this case the over-distention of the uterus with amniotic fluid caused inertia and paralysis of the muscular structure of the uterus, and thus prevented fatty degeneration from taking place in the placental site.

A very soft placenta is a frequent cause of adhesion, especially if it be thin, of large superficies, so as to be diffused over a considerable portion of the surface of the uterus. The contracting uterus does not readily throw off such a placenta, at least completely. Perhaps a large part may be expelled or withdrawn, and appear to be all; but a portion of a cotyledon remains behind; bleeding and irregular action of the uterus are kept up, until the hand is introduced and the offending substance removed.

A placenta succenturiata may occur and one lobe of it be left in the uterus. At a distance from the main body of the placenta, perhaps three or four inches or more from the margin, a mass of chorion-villi will be developed into this placental structure, and connected with the main body only by a few vessels. It resembles a lobe or cotyledon which has grown far away from the rest. Such an accidental or supernumerary placenta may very easily remain attached after the main body, which is complete in itself, has been removed. They measure about three or four inches in diameter.

It might be well to mention here the connection that exists between "milk-fever" and retention of portions of the placenta and clots in the uterus. Winckel, Hecker, Grunewaldt, Barker and D'Espine have entirely abolished milk-fever, and see in the febrile disturbances which sometimes appear when the function of lactation is being developed, only evidence that the system has absorbed a small dose of septic poison.

As far as my own experience goes, I have never seen a case of milk-fever occur in a patient where I was satisfied that the uterus was completely and thoroughly emptied and firmly contracted. But I have frequently seen it where clots and coagula remained in the uterus, undergoing decomposition and passing out with the lochia about the second or third day.

The consequences of partial retention of the placenta are—first, immediate, and second, remote.

1. Hemorrhage and spasmodic pain.

2. Decomposition of blood in the cavity of the uterus, and imprisonment of the products, by the placenta blocking up the orifice, constituting physometra. In this condition the uterus sometimes enlarges, becoming tympanitic.

3. Septicæmia from the absorption of the foul products.

4. Inflammation of the uterus and peritoneum, possibly from escape of the products of decomposition by the fallopian tubes into the peritoneal cavity.

5. Acute inflammation of the mammary gland.

The remote effects are:

Sub-involution, pelvic abscess, fungous degeneration of the mucous membrane of the uterus, polypi of the cavity and cervix, menorrhagia, the various displacements of the uterus, etc. In regard to the complete disappearance of the placenta by disintegration, liquefaction or absorption, there is a general expression of doubt among obstetrical authorities. Barnes says he does not believe it can be established on good evidence—he says he feels disposed to regard it in the same light as Velpeau regarded "vagitus uterinus" (the crying of the child in utero.) Since men of credit affirm that they have seen it, I believe it; but if I had seen it myself I should doubt.

In the *American Journal of Obstetrics*, Vol. x, p. 389, Dr. Trush, of Cincinnati, relates a very interesting case of retention and absorption of placenta, and in Vol. xi, of the same *Journal*, page 562, he reports that the same patient in her next labor had again complete retention of the placenta, which he and another physician were unable to remove.

He found in the right superior angle of the uterus, where the placenta of the first pregnancy had been located, a number of fibrous cords in a kind of net-work. He attempted to break them down, but found them very strong. He considered them as remnants of the first placenta, the more succulent structures having been removed by absorption, while a few of the most obdurate fibrous trabeculæ withstood this process.

In both of these cases, portions of the disintegrated placenta passed with the lochial discharge, which for several days was very offensive. There was no decided rise of temperature and no indication of septic poisoning.

In looking over the books on obstetrics, we find that the same advice has been handed down from one writer to another for the past century. They all say after the child is delivered and the placenta expelled, examine carefully the after-birth to see if it has all come away, and then secure contraction of the uterus. Now, as we have already seen, there may be two distinct placentæ, or a large portion of one may be left behind, and the most careful examination of what has been removed may fail to inform us whether or not any remains.

We are told that meddlesome midwifery is a dangerous thing; that we should leave everything to nature; that any handling or manipulations about the uterus at this time should be avoided. This same doctrine might be applied with as much reason to the surgeon who is called upon to treat a recent injury, and who contents himself with examining the foreign substance that he has removed instead of the wound itself, or to the lithotomist who examines the calculus that he has removed instead of the bladder, or to the oculist who merely examines the substance he has removed instead of the eye itself to know whether he has done his full duty.

When secondary hemorrhage comes on, or peritonitis, or mammary abscess occur, or any of the diseases to which the puerperal woman is liable, the thought at once presents itself, "Could it be possible that any portion of the placenta or any clots were left behind in the uterus to cause this trouble?" If we can answer this question with truthful assurance, we are at once relieved of a weight of anxiety, perplexity and doubt. Certainly no one can envy the position of an attendant in a case where placental remains have been expelled after causing dangerous complications, or the fate of one against whom perhaps a suit of mal-practice is brought where the uterus and retained portion of the placenta are presented in court as damaging evidence to his cause.

Although various plans are advised by different obstetric writers, the one known as Crede method accomplishes best the complete expulsion of the placenta and coagula, insuring at the same time firm contraction of the uterus. By manual compression the uterus is forced to cast the placenta by its own effort. If now, while one hand still grasps the contracting and expelling uterus, the other removes the placenta from the vagina, and then two fingers are introduced into the uterus, and while compression is still continued externally, the whole interior of the uterus is explored and every clot removed, we find by this means much firmer contractions are secured, hemorrhage is not likely to occur as it is when even a few clots remain. There are rarely any after pains, the lochial discharge ceases much sooner, involution of the uterus goes on more rapidly. Erosions and ulcerations of the cervix are not liable to occur, as there is no fœtid and decomposing lochial discharges to bathe these parts. If there is any laceration of the cervix or partial laceration of the perineum, they will generally heal without trouble. Subinvolution of the vagina, one of the most frequent causes of prolapsus uteri, is not liable to occur.

In concluding this subject, I cannot do better than to recall to your minds the following extract from Dr. Fordyce Barker's work on the puerperal diseases. He says:

"It is far from the truth to say that partial retention of the placenta always arises from the neglect or ignorance of the medical attendant, for this casualty has occurred in the hands of some of the ablest and most eminent obstetricians, who have reported numerous fatal cases of homorrhage from this cause. But I cannot impress upon you too strongly, in all cases where the artificial removal of the placenta is required, to exercise the greatest care to remove the whole of it, if this can be accomplished. In some cases of very close and intimate morbid adhesions, it may not be possible to accomplish this. But this I will say in unqualified terms—that every physician should know whether or not he has left a portion of the placenta behind, and he is justly censurable when he is ignorant on this point.—*Maryland Med. Jour.*

